# Seroprevalence of human T-cell lymphotropic virus and transfusion-transmitted viral hepatitis markers among hemodialysis and thalassemia patients in the South of Iran

**DOI:** 10.1186/s12879-026-14010-1

**Published:** 2026-07-13

**Authors:** Fatemeh Farshadpour, Zahra Avazzadeh, Fatemeh Dehghanian, Samad Akbarzadeh, Ali Movahed, Katayoun Vahdat, Manoochehr Makvandi, Narges Obeidi, Reza Taherkhani

**Affiliations:** 1 Department of Virology, School of Medicine, Bushehr University of Medical Sciences, Bushehr, Iran; 2 Department of Clinical Biochemistry, School of Medicine, Bushehr University of Medical Sciences, Bushehr, Iran; 3 The Persian Gulf Tropical Medicine Research Center, Bushehr University of Medical Sciences, Bushehr, Iran; 4 Virology Department, School of Medicine, Ahvaz Jundishapur University of Medical Sciences, Ahvaz, Iran; 5 Department of Hematology, School of Paramedicine, Bushehr University of Medical Sciences, Bushehr, Iran

**Keywords:** Human T-cell lymphotropic virus, Blood transfusion, *β-thalassemia*, Hemodialysis, Seroprevalence, Iran

## Abstract

**Background:**

This study was designed to determine the seroprevalence of HTLV among multi-transfused patients in the South of Iran.

**Methods:**

All patients with *β-thalassemia* major and those on regular hemodialysis from the cities of Dashtestan, Bushehr, Genaveh, Tangestan, and Kangan participated in this study. Serum samples were tested for HTLV-I/II antibodies by ELISA, and Western Blot confirmed seropositive samples. Moreover, hepatitis-related markers were assessed using serological assays.

**Results:**

Of 134 thalassemia patients, 6 cases (4.48%, 95% CI: 1.83%-9.91%) were positive for HTLV-I/II antibodies, all of which were confirmed by Western blot as HTLV-1. The hemodialysis patients and the controls were negative for HTLV-I/II antibodies. Of 6 HTLV-1 seropositive thalassemia patients, 4 cases were positive for anti-HCV antibodies (66.7%), and 2 cases were positive for anti-HEV IgG (33.4%). HTLV-1 seropositive thalassemia patients were negative for HBV infection criteria, while 5 of 6 patients (83.33%) had HBsAb in their samples. HTLV-1 seropositivity increased with age and was higher among single patients, female patients, residents of Kangan, patients receiving blood transfusions every two weeks, and Fars patients. Nevertheless, the seroprevalence of HTLV-1 was not statistically associated with marital status, gender distribution, place of residency, frequency of blood transfusion, age, ethnicity, or level of education.

**Conclusion:**

This study reports a relatively high seroprevalence of HTLV-1 among patients with *β-thalassemia* major, highlighting a high risk of HTLV-1 exposure in the thalassemia population. Therefore, an evaluation of introducing HTLV donor screening is recommended to reduce the transmission of HTLV-1 infection.

## Introduction

Human T-cell lymphotropic virus type-1 (HTLV-1) is a single-stranded, positive-sense RNA virus of the genus *Deltaretrovirus* in the family *Retroviridae* [1]. The clinical manifestations of HTLV-1 infection vary from lifelong asymptomatic infection to malignancies such as adult T-cell leukemia/lymphoma (ATL), and inflammatory disorders such as HTLV-1-associated myelopathy/tropical spastic paraparesis (HAM/TSP), alveolitis, uveitis, and infective dermatitis [2, 3]. HTLV-1, with 5 million to 10 million infected individuals, has a worldwide distribution [4]. However, the infection is more prevalent in endemic countries, including sub-Saharan Africa, Latin America, Japan, Romania, Iran, and Australia [1, 5]. HTLV-1 is transmitted through blood transfusion, intravenous drug abuse, organ/tissue transplantation, sexual intercourse, and breastfeeding [1, 5].

Transfusion of blood and blood products containing HTLV-infected lymphocytes is the most effective route of HTLV transmission [1], with a seroconversion rate of nearly 50% during whole-cell transfusions [3]. While most HTLV-1-infected patients remain asymptomatic for their lifetime, some cases might progress to life-threatening malignancies [1]. Based on ethnicity, 1% to 5% of HTLV-1-infected individuals are at risk of developing ATLL or HAM/TSP [2, 6]. Moreover, treatment options are not satisfactory [5]. Therefore, those patients requiring frequent transfusions are at increased risk of being infected and developing life-threatening conditions, especially in areas where there is no national screening program for HTLV-1 [1, 3].

Iran is an endemic country for HTLV-1 infection [7]. However, despite this endemicity and the importance of blood transfusion in HTLV transmission, multi-transfused patients are not screened for HTLV-1 infection. Because most infected individuals remain undiagnosed due to the asymptomatic nature of HTLV-1 infection, screening of blood transfusion recipients for HTLV-1 seems necessary. Information on the prevalence of HTLV among recipients of unscreened blood transfusions can help healthcare providers make informed decisions about the best management and prevention strategies for HTLV-1 infection in multi-transfused patients. Therefore, this study was designed to investigate the seroprevalence of HTLV among patients with *β-thalassemia* major and those on regular hemodialysis in the South of Iran.

## Subjects and methods

### Study design and population

All patients on regular hemodialysis and patients with *β-thalassemia* major attending the dialysis centers and the thalassemia centers of the Bushehr University of Medical Sciences were invited to participate in this study. Overall, 332 hemodialysis patients and 134 thalassemia patients from the cities of Dashtestan, Bushehr, Genaveh, Tangestan, and Kangan in southern Iran participated in this study. Moreover, 138 and 335 volunteers from out-patient subjects were enrolled as control groups for the thalassemia and hemodialysis cases, respectively. The controls were hospital visitors from the same cities as cases and did not have a history of blood transfusion, hemodialysis, or injecting drug abuse, and were matched in age, sex, and date of participation with the cases.

### Sampling and participant recruitment

All participants have signed written informed consent forms to use their data and blood samples for analysis and detection of HTLV infection. The socio-demographic data and medical information were obtained through interviews or from the subjects’ clinical records at hemodialysis and thalassemia centers or hospitals. This study was funded by the Deputy Research and Affairs of the Bushehr University of Medical Sciences with grant numbers 1401-3 and 1401-4. The Ethics Committee of the University approved this study with reference numbers IR.BPUMS.REC.1401.019 and IR.BPUMS.REC.1401.020. In addition, all methods were performed in accordance with the relevant guidelines and regulations.

### Serological and confirmatory assays

Serum samples of participants were tested for the presence of HTLV-I/II antibodies using enzyme-linked immunosorbent assay (ELISA) according to the manufacturer’s instructions (HTLV I&II Ab ELISA kit, DIA.PRO, Milan, Italy). The sensitivity and specificity of this ELISA kit were 100%. Furthermore, the repeatedly positive samples were confirmed by Western Blot (HTLV BLOT 2.4, MP Diagnostics, Singapore). Additionally, HTLV-1 seropositive samples were evaluated for the presence of anti-HCV antibodies, hepatitis B surface antigen (HBsAg), HBsAb, hepatitis B core antibody (HBcAb), HDV antibody, and anti-HEV antibodies according to the results of our previous studies [8–13]. The hepatitis serological marker data were obtained from the same participants and serum sample collection period as the current study, and these data were previously published in the cited studies.

### Statistical analysis

The data were analyzed by the SPSS 17 package program (SPSS Inc., Chicago, IL, USA) and were presented as frequencies, percentages, or mean ± standard deviation (SD). Student’s t-test and χ2 test or Fisher’s exact test were used to analyze and compare the data between HTLV-positive and HTLV-negative patients, and *P* values less than 0.05 were considered statistically significant.

## Results

### Demographic and clinical characteristics of the study population

In this study, 134 patients with *β-thalassemia* major (54 males and 80 females), including 103 patients from Bushehr, 14 patients from Tangestan, 14 patients from Dashtestan, and 3 patients from Kangan city, and 332 hemodialysis patients (177 males and 155 females), including 228 patients from Bushehr, 58 patients from Dashtestan, and 46 patients from Genaveh city, were enrolled. The mean age ± SD of the participants was 59.23 ± 14.07 years with a range of 21–87 years for the hemodialysis patients and 27.4 ± 10.84 years with a range of 1–49 years for the thalassemia patients. The majority of hemodialysis patients were in the age groups 50–59 years and 60–69 years (52.4%), male (53.3%), residents of Bushehr (68.7%), Fars (93.1%), under diploma (80.7%), and more than one year under dialysis (81.9%). The majority of thalassemia patients were in the age groups 20–29 years and 30–39 years (62.7%), female (59.7%), residents of Bushehr (76.9%), Fars (91.0%), single (91.0%), and under diploma (51.5%), and had blood transfusion once a month (50.7%) (Table 1).

**Table 1:** The seroprevalence of HTLV-1 according to socio-demographic characteristics and quantitative variables among patients with *β-thalassemia* major in the South of Iran

	No. of all participants (%): 134 (100%)	No. of HTLV Ab negative subjects (%):128 (95.5%)	No. of HTLV Ab positive subjects (%):6 (4.5%)	*P*-Value
**Age groups (years)**				**0.213**
< 10	9 (6.7%)	9 (100.0%)	0 (0.0%)	
10–19	24 (17.9%)	24 (100.0%)	0 (0.0%)	
20–29	47 (35.1%)	45 (95.7%)	2 (4.3%)	
30–39	37 (27.6%)	33 (89.2%)	4 (10.8%)	
> 39	17 (12.7%)	17 (100.0%)	0 (0.0%)	
**Gender**				**0.401**
Female	80 (59.7%)	75 (93.8%)	5 (6.3%)	
Male	54 (40.3%)	53 (98.1%)	1 (1.9%)	
**City**				**0.078**
Bushehr	103 (76.9%)	99 (96.1%)	4 (3.9%)	
Dashtestan	14 (10.4%)	14 (100.0%)	0 (0.0%)	
Tangestan	14 (10.4%)	13 (92.9%)	1 (7.1%)	
Kangan	3 (2.2%)	2 (66.7%)	1 (33.3%)	
**Ethnicity**				**0.734**
Fars	122 (91.0%)	116 (95.1%)	6 (4.9%)	
Afghan	3 (2.2%)	3 (100.0%)	0 (0.0%)	
Arab	9 (6.7%)	9 (100.0%)	0 (0.0%)	
**Marital Status**				**0.563**
Single	122 (91.0%)	116 (95.1%)	6 (4.9%)	
Married	12 (9.0%)	12 (100.0%)	0 (0.0%)	
**Frequency of Blood Transfusion**				**0.460**
Once every two weeks	7 (5.2%)	6 (85.7%)	1 (14.3%)	
Once every three weeks	27 (20.1%)	26 (96.3%)	1 (3.7%)	
Once a month	68 (50.7%)	65 (95.6%)	3 (4.4%)	
As needed	9 (6.7%)	8 (88.9%)	1 (11.1%)	
Unknown	23 (17.2%)	23 (100.0%)	0 (0.0%)	
**Education**				**0.001**
Under diploma	69 (51.5%)	69 (100.0%)	0 (0.0%)	
Diploma	53 (39.5%)	50 (94.3%)	3 (5.7%)	
Upper diploma	12 (9.0%)	9 (75%)	3 (25%)	
**ALT level (IU/L)**				**0.332**
< 20	49 (36.6%)	46 (93.9%)	3 (6.1%)	
20–40	32 (23.9%)	29 (90.6%)	3 (9.4%)	
41–80	20 (14.9%)	20 (100.0%)	0 (0.0%)	
> 80	9 (6.7%)	9 (100.0%)	0 (0.0%)	
Unknown	24 (17.9%)	24 (100.0%)	0 (0.0%)	
**AST level (IU/L)**				**0.280**
< 20	29 (21.6%)	27 (93.1%)	2 (6.9%)	
20–40	46 (34.3%)	42 (91.3%)	4 (8.7%)	
41–80	28 (20.9%)	28 (100.0%)	0 (0.0%)	
> 80	7 (5.2%)	7 (100.0%)	0 (0.0%)	
Unknown	24 (17.9%)	24 (100.0%)	0 (0.0%)	

### HTLV seropositivity rates

Of 134 thalassemia patients, 6 cases (4.48%, 95% CI: 1.83%-9.91%) were positive for HTLV I/II antibodies, all of which were confirmed by Western blot. HTLV seropositive samples were detected as HTLV-1, while HTLV-2 antibodies were not found in the sample. The hemodialysis patients (0.00%, 95% CI: 0.00%-1.43%) and the controls were negative for HTLV I/II antibodies.

### Risk factor analysis

HTLV-1 seropositive thalassemia patients were 25–39 years old. The majority of HTLV-1 seropositive thalassemia patients were in the age group 30–39 years (10.81%, 95% CI: 4.28%-24.71%), followed by the age group 20–29 years (4.26%, 95% CI: 1.18%-14.26%), whereas the age groups < 10 years, 10–19 years, and > 39 did not show HTLV-1 seropositivity (Table 1). Moreover, HTLV-1 seropositive thalassemia patients had a higher mean age (33.00 ± 6.45) than HTLV-1 seronegative thalassemia patients (27.13 ± 10.94), but this difference was not statistically significant (*P* = 0.196). Regarding ethnicity, HTLV-1 antibodies were detected in 4.9% of Fars thalassemia patients, whereas Afghan and Arab thalassemia patients were HTLV-1 seronegative (*P* = 0.734). The seroprevalence of HTLV-1 among male and female thalassemia patients was 1.9% and 6.3%, respectively (*P* = 0.401). Moreover, the seroprevalence of HTLV-1 was higher among single patients with *β-thalassemia* (4.9%), residents of Kangan (33.3%), patients requiring blood transfusions every two weeks (14.3%), and upper diploma patients (25.0%). Nevertheless, the seroprevalence of HTLV-1 among thalassemia patients was not statistically associated with marital status, gender distribution, place of residency, frequency of blood transfusion, age, ethnicity, or level of education (Table 1).

### Hepatitis viruses’ serological markers

Of 6 HTLV-1 seropositive thalassemia patients, 4 cases were positive for anti-HCV antibodies, and 2 cases were positive for anti-HEV IgG. All HTLV-1 seropositive patients were negative for HBsAg, HBcAb, and HDV antibody and had normal ALT and AST levels. Notably, five HTLV-1 seropositive thalassemia patients had HBsAb in their samples.

## Discussion

HTLV-1 is endemic in Iran [7] and is effectively transmitted through unscreened blood transfusion [1]. In the absence of prophylactic measures and satisfactory treatment options [5], screening of blood and blood products for HTLV-1 before transfusion is the best way to control this viral infection. Nevertheless, HTLV-1 screening is not routinely implemented in blood banks in the South of Iran. Information on the prevalence of HTLV-1 among multi-transfused patients can convince health authorities to implement HTLV-1 screening of donated blood in the blood transfusion organization of this region. Therefore, we evaluated the seroprevalence of HTLV-1 infection among multi-transfused patients in the South of Iran and found an HTLV-1 seropositivity rate of 4.48% in *β-thalassemia* major patients. However, the hemodialysis patients were negative for HTLV I/II antibodies.

The HTLV-1 seroprevalence of 4.48% among thalassemia patients is higher than that reported among blood donors (0.20%) and the general population (2.5% in endemic regions and 0.07% to 1.8% in non-endemic regions) in Iran [14]. Moreover, the HTLV-1 seroprevalence reported in this study is much greater than that identified in non-thalassemic controls (0.00%) and blood donors (0.013%) [15] living in this region. According to the results of this study, thalassemia patients are at high risk of exposure to HTLV-1 in this region, probably due to unscreened blood transfusions. However, the cross-sectional design of the study limits causal inference. Therefore, this study cannot determine the causal relationship between blood transfusion and HTLV transmission. Prospective cohort studies are required to evaluate the potential role of blood transfusion in HTLV acquisition among thalassemia patients in the South of Iran. There is an increase in the seroprevalence of HTLV-1 infection among thalassemia patients over the last two decades in the South of Iran, so that the seroprevalence of HTLV-1 has increased from 3.08% in 2003 [16] to 4.48% in 2025. Considering the high seroprevalence and the increasing trend of HTLV-1 infection among thalassemia patients, an evaluation of introducing HTLV donor screening is recommended in the South of Iran. However, the other potential routes of HTLV transmission, including sexual intercourse, intravenous drug abuse, and breastfeeding, should also be considered.

The HTLV-1 seroprevalence identified in this study is higher than those reported among patients with *β-thalassemia* major in the other parts of Iran, 1.6% in Sistan-Bluchistan (South-East of Iran) [17], 2.17% in Kordestan (West of Iran) [18], 3.3% in Isfahan (Center of Iran) [19] and 3.45% in Kermanshah (West of Iran) [20] but lower than 6.11% in Mashhad (North-East of Iran) [21], 6.3% in Tehran (Center of Iran) [22], 6.8% in Charmahal-va-Baktiari (Center of Iran) [23] and 6.9% in Mazandaran (North of Iran) [24]. The HTLV-1 seroprevalence reported in this study is slightly higher than the overall prevalence of HTLV-1 among thalassemia patients in Iran (4.1%) [3]. It is also higher than that reported among thalassemia patients in some other countries, 1.3% in the USA [25] and 1.07% in Greece [26]. In this study, all hemodialysis patients were negative for HTLV antibodies. High seroprevalence of HTLV-1 has been reported among hemodialysis patients in Neyshabour (14.5%) [27] and Charmahal-va-Baktiari (6.54%) [23]. According to a recent meta-analysis study, the overall seroprevalence of HTLV among hemodialysis patients in Iran is 2.37% [7]. High seroprevalence of HTLV has been reported among hemodialysis patients in Iraq (46.2%) [28]. The HTLV-1 seropositivity rate of 2.48% was identified among hemodialysis patients in Brazil [29], while HTLV antibodies were not detected among hemodialysis patients in Greece [30]. Differences in the burden and endemicity of HTLV infection and transfusion safety measures in various geographical regions can explain these variations in HTLV prevalence in different studies.

In this study, the seroprevalence of HTLV-1 was higher among single patients, female patients, residents of Kangan, those receiving blood transfusions every two weeks, and Fars patients. Moreover, HTLV-1 seropositivity increased with age, ranging from 4.26% in *β-thalassemia* patients 20–29 years old to 10.81% in *β-thalassemia* patients 30–39 years old, suggesting cumulative exposure to events that increase the risk of acquiring HTLV over time, such as blood transfusions. Nevertheless, the seroprevalence of HTLV-1 among *β-thalassemia* patients was not statistically associated with marital status, gender distribution, place of residency, frequency of blood transfusion, age, ethnicity, or level of education. Those studies in Kermanshah, Mazandaran, and Tehran reported a higher frequency of HTLV-1 among male patients [20, 22, 24]. Previous studies from Iraq and Neyshabour (North-East of Iran) reported a significant association between higher HTLV-1 seroprevalence and older ages in hemodialysis patients [27, 28]. In a study from Brazil, HTLV seroprevalence among hemodialysis patients was significantly associated with age, history of blood transfusion, and marital status [29].

In this study, 4 of 6 HTLV-1 seropositive patients (66.7%) were positive for anti-HCV antibodies. The high percentage of HTLV-1 and HCV serological markers indicates similar routes of transmission for these viral infections. This issue should be considered in the prevention and management of viral infections among multi-transfused patients. In a study from Tehran, 44.8% of HTLV-1 seropositive thalassemia patients were co-infected with HCV [31]. In another study from Iraq, 13.9% of hemodialysis patients were co-infected with HTLV and HCV, and 3.8% were co-infected with HTLV and HBV [28]. In this study, 2.98% of thalassemia patients were positive for both HTLV-1 and HCV antibodies. It is noteworthy that HTLV-1 has immune-modulating effects that might worsen HCV infection outcomes [32]. On the other hand, HTLV and HBV co-infection seems to influence the clearance of HBV. In a recent study from Australia, HTLV-1-positive patients showed a high prevalence of HBV infection (67.6%), possibly due to a decreased rate of HBV clearance or persistent HBV infection in HTLV-1-infected patients [32]. In our study, HTLV-1 seropositive thalassemia patients were negative for HBV infection criteria, while 5 of 6 HTLV-1 seropositive patients (83.33%) had HBsAb in their samples. Moreover, 2 of 6 HTLV-1 seropositive patients (33.4%) were positive for anti-HEV IgG.

This study reports a relatively high seroprevalence of HTLV-1 among patients with *β-thalassemia* major in the South of Iran, suggesting the possibility of HTLV transmission via blood transfusion in this region. However, other sources of HTLV infection, such as transmission via intravenous drug use (IDU), were not evaluated in this study. Since there was no information on the history of IDU in the patients’ records, and some participants were unwilling to answer questions about IDU. The presence of the viral genome was not investigated in the serum samples of HTLV-1 seropositive participants. Therefore, the precise prevalence of HTLV-1 infection, the molecular epidemiology of HTLV-1 infection, and proviral load analysis remained unknown in this study. The cross-sectional design of the study prevents follow-up of the participants. Therefore, the temporal assessment of infection acquisition was impossible among the study population. Moreover, donor tracing was not possible due to the lack of information in the records of blood banks. As another limitation, we had no information on HIV prevalence among the study population. Therefore, the status of HIV infection remained unknown among the HTLV-1-positive patients. The sample size of HTLV-1 seropositive cases is very small, and this is the major limitation of the current study. Therefore, statistical comparisons between HTLV-positive (*n* = 6) and HTLV-negative (*n* = 128) patients suffer from low statistical power. Accordingly, multivariable analysis was not performed because of the limited number of HTLV-1 seropositive cases.

## Conclusion

This study reports HTLV-1 seroprevalence of 4.48% among patients with *β-thalassemia* major in the South of Iran. The observed HTLV-1 seroprevalence among multi-transfused *β-thalassemia* patients suggests the possibility of transfusion-transmitted infection and supports an evaluation of introducing HTLV donor screening. However, this study cannot determine the causal relationship between blood transfusion and HTLV transmission. Therefore, other potential routes of HTLV transmission, including sexual intercourse, intravenous drug abuse, and breastfeeding, should also be considered. Moreover, given the asymptomatic nature of HTLV-1 infection and risk of developing life-threatening disorders, training programs and effective precautions are also advisable to reduce viral transmission dynamics.

## Data Availability

All relevant data are within the paper.

## Abbreviations

HTLV-1: Human T-cell lymphotropic virus type-1
ATL: Adult T-cell leukemia/lymphoma
HAM/TSP: HTLV-1-associated myelopathy/tropical spastic paraparesis
ELISA: Enzyme-linked immunosorbent assay
HBsAg: Hepatitis B surface antigen
HBcAb: Hepatitis B core antibody
SD: Standard deviation
OR: Odds ratio
95% CI: 95% Confidence intervals
